# Trait Impulsivity and Anhedonia: Two Gateways for the Development of Impulse Control Disorders in Parkinson’s Disease?

**DOI:** 10.3389/fpsyt.2016.00091

**Published:** 2016-05-30

**Authors:** Jean-Luc Houeto, Robin Magnard, Jeffrey W. Dalley, David Belin, Sebastien Carnicella

**Affiliations:** ^1^Service de Neurologie, CIC-INSERM 1402, CHU de Poitiers, Université de Poitiers, Poitiers, France; ^2^INSERM U1216, Grenoble Institut des Neurosciences (GIN), University Grenoble Alpes, Grenoble, France; ^3^Department of Psychology, University of Cambridge, Cambridge, UK; ^4^Department of Psychiatry, University of Cambridge, Cambridge, UK; ^5^Department of Pharmacology, University of Cambridge, Cambridge, UK

**Keywords:** Parkinson’s disease, impulse control disorders, impulsivity, apathy, depression, anxiety, dopaminergic nigrostriatal system, D_2/3_ dopamine receptors

## Abstract

Apathy and impulsivity are two major comorbid syndromes of Parkinson’s disease (PD) that may represent two extremes of a behavioral spectrum modulated by dopamine-dependent processes. PD is characterized by a progressive loss of dopaminergic neurons in the substantia nigra pars compacta to which are attributed the cardinal motor symptoms of the disorder. Dopamine replacement therapy (DRT), used widely to treat these motor symptoms, is often associated with deficits in hedonic processing and motivation, including apathy and depression, as well as impulse control disorders (ICDs). ICDs comprise pathological gambling, hypersexuality, compulsive shopping, binge eating, compulsive overuse of dopaminergic medication, and punding. More frequently observed in males with early onset PD, ICDs are associated not only with comorbid affective symptoms, such as depression and anxiety, but also with behavioral traits, such as novelty seeking and impulsivity, as well as with personal or familial history of alcohol use. This constellation of associated risk factors highlights the importance of inter-individual differences in the vulnerability to develop comorbid psychiatric disorders in PD patients. Additionally, withdrawal from DRT in patients with ICDs frequently unmasks a severe apathetic state, suggesting that apathy and ICDs may be caused by overlapping neurobiological mechanisms within the cortico-striato-thalamo-cortical networks. We suggest that altered hedonic and impulse control processes represent distinct prodromal substrates for the development of these psychiatric symptoms, the etiopathogenic mechanisms of which remain unknown. Specifically, we argue that deficits in hedonic and motivational states and impulse control are mediated by overlapping, yet dissociable, neural mechanisms that differentially interact with DRT to promote the emergence of ICDs in vulnerable individuals. Thus, we provide a novel heuristic framework for basic and clinical research to better define and treat comorbid ICDs in PD.

Idiopathic Parkinson’s disease (PD) is a neurodegenerative disorder, resulting mainly from the loss of dopaminergic (DA) neurons in the substantia nigra pars compacta (SNc) and characterized by tremor, rigidity, and bradykinesia (1). Although classically defined by the resulting motor symptoms, this neurological disorder is also associated with a plethora of non-motor manifestations (2, 3). These non-motor symptoms, some of which predating the occurrence of overt motor impairment (2, 4–7), are now increasingly recognized to contribute detrimentally to the patients’ quality of life (6, 8). They include sensory (such as pain and a loss of smell or hyposmia) and autonomic dysfunctions, alteration of sleep, as well as cognitive disturbances (2, 4, 5, 7). Neuropsychiatric symptoms in PD range from dramatic deficits in hedonic processes, including a decrease in motivated behaviors (apathy) and mood/affective impairments, to impulse control disorders (ICDs) (5, 9–12). While the former are mostly expressed during the reduction, or withdrawal, of dopamine (DA) replacement therapy (DRT), the latter are considered as frequent complications of DRT [e.g., Ref. (10–12)]. ICDs include a heterogeneous group of behavioral addictions, such as pathological gambling and hypersexuality, as well as punding and the compulsive misuse of dopaminergic medication (13). Despite dramatic social, occupational, and familial impacts, the etiological and pathophysiological substrates of ICDs in PD remain unclear (14, 15). This may be partly due to the paucity of studies that have attempted to operationalize these comorbid symptoms in experimental animals, which, through longitudinal studies uniquely offered by preclinical models, would help identify the psychological, behavioral, and neural mechanisms subserving individual vulnerability to develop ICDs in PD patients.

Here, after a description of the phenomenology of ICDs and their associated risk factors, we discuss the evidence that high impulsivity trait and anhedonia-related behaviors play important contributory roles to the development and the expression of ICDs in PD. We next capitalize on the wealth of literature on the neurobiological mechanisms of impulsivity and anhedonia-related behaviors in PD to suggest that impulse control and hedonic/motivational deficits may represent distinct prodromic gates for the development of ICDs, through compulsive enhancement seeking and self-medication failure, respectively. Based on these hypotheses, we propose a novel heuristic framework to implement relevant preclinical studies and improve the management of comorbid psychiatric symptoms in PD.

## Phenomenology of ICDs

### What Are ICDs?

Impulse control disorders reported in patients with PD include pathological gambling [now termed as gambling disorder (16)], hypersexuality, compulsive shopping, and binge eating, with or without the presence of excessive creativity [e.g., Ref. (17)]. These aberrant behaviors reflect the maladaptive nature of the preoccupations of the patients, their inability to control their urges or impulses, which trigger other compulsive behaviors, such as lying or stealing. On the severity scale of these behaviors, which should not occur exclusively within a manic episode, pathological state is defined as the presence of clear distress or interference with social, financial, or occupational functioning (9). Impulsive–compulsive behaviors or “behavioral addictions” (18–20), regardless of their clinical expressions (see below), impinge on the quality of life of the patients and can have serious or even dramatic familial, social, and economic consequences (9, 21–24).

At the clinical level, gambling behavior includes different activities like playing cards for money, betting on horses, dogs, or sports games, playing the stock or commodities market, buying lottery tickets, playing bingo, as well as gambling at a casino, with a marked preference for playing slot machines or gambling on internet, suggesting a bias toward immediate gratification, and repetitive motor acts (9, 13, 25). Hypersexuality include inappropriate or excessive requests of sex from a spouse or a partner, permanent preoccupation with pornography, telephone sex lines, masturbation, or compulsive promiscuity and paraphilia (26). Frequently, after an orgasm the patient does not reach satisfaction, and the continuous necessity of performing, or thinking about, sex may ultimately generate anxiety and frustration. Compulsive shopping is more frequently present in women than in men and involves the excessive necessity to buy anything, often unnecessary things with severe financial consequences (9, 27, 28). Binge eating is defined as the compulsive ingestion of a large amount of food in a short period of time, and compulsive eating is described as an abnormal ingestion of food in excess, during protracted periods of time, with no necessity to alleviate hunger. The first description of punding was made in the 1970s and as a behavior observed in amphetamine addicts (29). Punding is a complex stereotyped behavior characterized by an intense fascination with repetitive, excessive non-goal-oriented manipulation (30). It can be simple (i.e., manipulating objects or instruments, sorting common objects) or complex hobbyism, such as hoarding, gardening, cleaning, singing, writing, pointless driving or walkabouts, and engaging in extended monologs devoid of content (31–33). The behavior over which control is lost in punding has been shown to be related to previously learned professional skills, as for instance, an accountant is more likely to shuffle paper (33).

Dopamine dysregulation syndrome (DDS), also termed hedonistic homeostatic dysregulation syndrome (34), was recently recognized as a consequence of compulsive misuse of DRT, beyond the dose needed to control motor disability, with patients fulfilling criteria for addiction. This disorder, clinically characterized by walkabouts and marked fluctuations in mood and psychosis, is present in 4% of PD patients with severe levodopa-induced dyskinesias and ICDs (35–37).

The association between dopaminergic medications and ICDs in PD is now well established. Indeed, the prevalence of ICDs has been found to be similar in newly diagnosed, untreated PD patients and healthy controls, but increases significantly under DRT (23, 24, 38–41). Although the frequency of ICDs varies and depends on the evaluation method and scales used, a multicentre cross-sectional study including more than 3000 PD patients, reported an overall prevalence of ICDs of 13.6% with 3.9% of patients having two or more ICDs (42). Interestingly, ICDs appears more common in patients treated with dopaminergic agonists than without (i.e., only levodopa medication). Indeed, the prevalence rate of ICDs in treated PD patients in UK and US clinics is considered to be 7% and up to 17% in PD patients without and with dopaminergic agonists, respectively (42). Specifically, 7.2% of patients with dopaminergic agonist suffered from compulsive buying against 2.9% without, 6.4% from pathological gambling against 2.3%, 5.6% from binge eating disorder against 1.7%, and 4.4% from compulsive sexual behavior against 1.7% (42). These prevalence rates, much higher than those observed in the general population, suggest that DRT, and especially dopaminergic agonists, probably in interaction with the pathophysiological substrates of PD, facilitate the emergence of impulsive/compulsive spectrum disorders (21, 43, 44).

Although the psychological, neural, and cellular substrates of vulnerability to ICDs in PD remain unknown, major risk factors have been identified. They are mostly related to early onset of PD in men, who are unmarried, with comorbid smoking habits, family history of gambling problems, and alcohol abuse (42). In these patients, ICDs were associated with high levels of novelty seeking, impulsivity, and depressed mood. They were related not only to treatment with DA receptor agonists or high dose of levodopa, but also to high-frequency subthalamic stimulation [(45, 46); but see Ref. (47)]. This neurosurgical treatment can also induce acutely hypomania [reviewed in Ref. (48)], thereby pointing to the role of this nucleus in impulsivity and the pathophysiology of ICDs (49, 50).

This non-exhaustive description of the epidemiology and phenomenology of ICDs emphasizes how much PD patients with ICDs resemble, in terms of neurobehavioral complications following DRT, those who suffer from drug addiction (19, 20, 51–55). This is consistent with the conceptualization that PD patients with ICDs – as opposed to those without – might share with drug addicts some psychological, neural, cellular, and genetic factors of vulnerability to impulsive/compulsive behaviors (19, 20, 56–58). This is further supported by the fact that relative to PD patients without ICDs, PD patients with ICDs score higher on measures of depression state and trait, aggressiveness, and anxiety and display higher novelty seeking, impulsivity trait, and impulsive choices, thereby suggesting that impulsivity and anhedonia-related syndrome are two core components of the pathophysiology of ICDs (10, 39, 59, 60).

### Impulsivity as a Core Symptom of ICDs

Impulsivity is a multi-faceted entity consisting of maladaptive behavior and characterized by poorly conceived, prematurely expressed, unduly risky, or inappropriate actions often resulting in undesirable consequences (61). Impulsivity has been recently subdivided into two major processes linked to different neural network and activated by distinct experimental paradigm: cognitive impulsivity and motor impulsivity (61). Cognitive impulsivity often refers to an inability to tolerate delays of reinforcement and, therefore, prefer immediate smaller rewards over distant larger ones. It can also refer to altered decision making, risk taking – under stable probabilistic contingencies (explicit risk taking) or ambiguous risk taking in which the subject is unaware of the probabilistic contingencies, perception of time (i.e., delay between the choice and the reception of the reward), and reversal learning (i.e., inability to reproduce behaviors that lead to positive outcome and to extinguish those that lead to negative outcomes) (61, 62). On the other hand, motor impulsivity refers to the ability to withhold an inappropriate response, the ability to stop an ongoing inappropriate response or, operationally, the speed with which we can inhibit an action that has, as a requisite of the task, become habitual (61, 62).

#### Cognitive Impulsivity in PD

Compared to PD patients without ICDs, PD patients with gambling disorder showed impaired decision-making (i.e., poorer performances on the Iowa Gambling Task) and cognitive impulsivity (preference for immediate over future, larger, rewards) (63, 64), both being key features of cognitive deficits in drug addicts (59, 61). In delay discounting tasks, PD patients with ICDs made more impulsive choices, with a reduced reaction time, than non-ICD PD patients, an effect further enhanced by DRT [e.g., Ref. (65)]. Thus, impulsive choice appears as a core symptom of ICDs, which is exacerbated by dopaminergic treatment in vulnerable PD patients. Neuroimaging studies have further suggested an increased impulsivity state in PD patients with ICDs. For instance, when submitted to gambling-related cues, alternating with neutral stimuli and rest periods, PD patients with gambling disorder displayed, relative to PD controls, abnormal activation of bilateral anterior cingulate cortex (ACC), medial and superior frontal gyri, and precuneus, right inferior parietal lobule and ventral striatum, areas implicated in impulse control (66). The over-activation of cingulate cortex and ventral striatum in PD/gamblers patients is again similar to that reported in addicted patients submitted to a drug craving situation (67). Another study, using [^11^C]-raclopride to compare D_2_ DA receptor availability during a control and a gambling task in two groups of PD patients, one with and the other without gambling disorder, both treated with dopamine agonists, found that patients with gambling disorder had increased release of dopamine in the ventral striatum during the gambling task [(68); see also Ref. (69)]. Similar heightened response of reward circuitry to heterogeneous reward-related visual cues were observed in PD patients with single or multiple co-occuring ICDs relative to PD controls without (70, 71). These results likely reflect an inappropriate reward response or abnormal striatal DA function, suggestive of maladaptive plasticity processes that could lead to defective top-down inhibitory control (70, 72).

#### Motor Impulsivity in PD

Relative to motor impulsivity, PD patients perform poorly on measures of response inhibition (62, 73). Of major interest, one of the most effective treatments for the motor symptoms of PD, namely, deep brain stimulation, exacerbates motor impulsivity both in humans [e.g., Ref. (74)] and rats (50, 75). Thus, subthalamic nucleus-DBS (STN-DBS) causes blood flow changes in ACC that correlate with a change in response inhibition, suggesting that the more DBS alters the function of inhibitory control-related cortical structures, the more it exacerbates impulsivity (76). This was further confirmed by the recent evidence that STN-DBS impairs response inhibition, measured as a greater number of errors during No-Go trials, these behavioral deficits being associated with reduced activation, as measured as H_2_^(15)^O positron emission tomography (PET)-based blood flow, in areas, such as the left premotor cortex, pre-supplementary motor area, dorsal ACC, and inferior frontal cortex (77). These areas are thought to subserve retroactive response inhibition in which a stop-stimulus must be processed and acted upon in order for the inhibition to be successful, in agreement with other recent report (78). Together with the recent evidence that the dopaminergic agonist pramipexole marginally disrupted response inhibition by activating the left lateral prefrontal cortex, sparing motor impulsivity measured in a Go–No-Go task [(79); see also Ref. (74)], these data suggest a double dissociation between DRT and STN-DBS on impulsivity, the former potentially influencing circuits responsible for impulsive choices, whereas the latter may alter circuits subserving impulsive actions.

### The Neurobiological Mechanisms of Impulsivity in PD

#### Loss of Dopaminergic Neurons May Promote Impulsivity in PD Patients

Although impulsive behaviors are aggravated under DRT, impulsivity levels may be overall higher in PD patients than in the general population, irrespective of treatment. Indeed, the higher level of impulsive choices displayed by PD patients without ICDs in an intertemporal choice task was not reduced during “off” medication periods (80). This might be due to a short withdrawal period, but similar results were observed in *de novo*, non-medicated patients (81), suggesting, as previously mentioned, that high impulsive choices are made by PD patients independently of the medication status, leading to the hypothesis that the loss of dopaminergic tone within the nigrostriatal pathway contributes to exacerbate impulsivity (82).

The neural and cellular substrates of impulsivity have been broadly defined using a combination of preclinical and clinical studies [for review, see Ref. (61, 83–85)]. Nevertheless, in general, attempts to recapitulate the co-occurrence of ICDs in PD using experimental animals have not been met with unequivocal success. Thus, although dopamine-depleting, 1-methyl-4-phenyl-1,2,3,6-tetrahydropyridine (MPTP) administration in non-human primates increases reaction times for delayed rewards, suggestive of increased impulsivity, this effect may be confounded by the non-specific motor-impairing effects of MPTP (86). Furthermore, in rats, 6-OHDA lesions of the nigrostriatal dopaminergic system reduce their tolerance for delayed certain rewards, but not uncertain rewards (87, 88).

**Figure 1:**
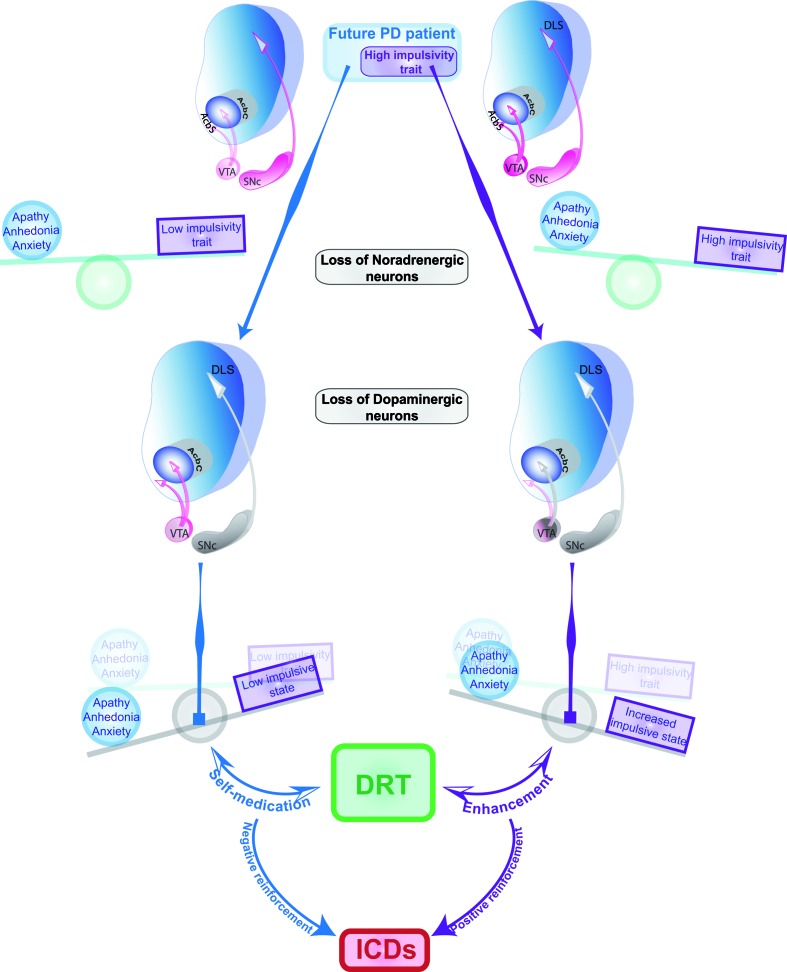
**Dual etiopathogenic pathways toward the development of ICDs in Parkinson’s disease**. ICDs may represent a common final state in vulnerable PD patients reflecting two independent mechanisms, whereby premorbid vulnerability factors interact with the neurodegenerative process to facilitate DRT-induced compulsive behaviors. Specifically, in some individuals with premorbid anxiety and depressive traits, potentially reflecting early alterations in the dopaminergic and noradrenergic system, dopaminergic loss within the nigrostriatal system, which, together with the loss of noradrenergic neurons of the locus coeruleus, contribute to the emergence of anhedonia-related states and behaviors, for which patients attempt to self-medicate with DRT. Compulsive DRT use in this case is supported by negative reinforcement mechanisms and contributes, as it has been suggested to be the case in drug addiction, to worsen the hedonic allostatic state, which, in the context of ICDs, is brought about by neurodegeneration. In other vulnerable patients, pre-existing high impulsivity, underlined by exacerbated dopaminergic activity in the NAc, may increase the susceptibility of dopaminergic neurons in the VTA to neurodegeneration. Thus, in patients with premorbid impulsivity, previously shown to predict an increased vulnerability to develop compulsive cocaine use, DRTs may facilitate the development of ICDs through a positive reinforcement mechanism. This would rely on the influence of DRT over hypodopaminergic mesolimbic and nigrostriatal pathways, by which DRT trigger an enhancement-seeking drive in the patient. DRT use will, therefore, not only further weaken the inhibitory control of these individuals but would also impinge on the imbalance between the functions of the ventral and the dorsal striatum, so that the enhancement seeking becomes rigid and compulsive. These two putative psychobiological processes should not be viewed as antagonistic but rather as two pathological mechanisms that will interact synergistically to weaken the control of the motivational and reward-related system and thereby facilitate the development of ICDs after repeated and excessive stimulation with DRT.

Taken together, these data support the hypothesis that dopaminergic cell loss within the nigrostriatal pathway contributes to increase impulsivity in PD (Figure 1). This has been further supported by PET studies showing not only a downregulation of D_2_/D_3_R binding in the caudate and putamen of PD patients that may be caused either by chronic DRT or the progressive decline of DA SNc neurons [(89); but see Ref. (90)] but also by a potential decrease in D_3_R binding in the ventral striatum of early-stage drug naive PD patients (91). Interestingly, a greater decrease in ventrostriatal D_3_R-binding in PD patients has been recently associated with the occurrence and the severity of ICDs (90). Additionally, in preclinical models of PD, bilateral DA denervation of the nigrostriatal system has been associated with an increase in DA levels in the nucleus accumbens (92). Although it deserves further investigation, such unique combination of diminished striatal D_2_/D_3_ receptors levels and increase in mesolimbic DA tone, both hallmark features of high impulsivity trait in humans and rats (93–96), may contribute to an increased propensity to develop impulsive behaviors in PD.

#### Dopaminergic Medication Enhanced Impulsivity in PD Patients

Nevertheless, dopaminergic medication does appear to be a key element in the development of ICDs in a subset of PD patients (13, 42, 97). The neural etiology of this interaction is unclear, but may have its origins either in an overdose of the mesolimbic system by DRT and/or in the extent of dopamine depletion in the nigrostriatal and mesolimbic systems (Figure 1) (98). Thus, PD initially involves dopamine depletion that is restricted mainly to the dorsal striatum, whereas for more advanced stages of the disease, dopamine depletion progressively involves the ventral striatum as well (99, 100). This may explain the apparent paradox why l-DOPA both improves and impairs distinct cognitive functions in PD patients, as this presumably depends on the precise extent of dopaminergic pathology in the striatum (101, 102). The hypothesis that l-DOPA and other dopaminergic medications acutely lead to an “overdose” of the relatively spared ventral striatum, and consequently the emergence of ICDs, is compatible with evidence that trait impulsivity is associated with increased activity of the mesolimbic system and the associated decreased D_2_/D_3_ dopamine receptor-binding in the ventral striatum in both humans and rats (93–96).

On the other hand, aberrantly increased activity of the mesolimbic system in highly impulsive individuals may contribute to an increased sensitivity of dopaminergic neurons of the ventral tegmental area (VTA) to dopamine-dependent oxidative stress and the neurodegenerative process in PD, thereby, influencing the pattern of dopaminergic denervation (Figure 1) so that DRT may interfere in these individuals with a partially denervated mesolimbic system that has lost the influence of phasic dopamine transmission. Thus, in highly impulsive individuals, the intact component of the mesolimbic system would be hyperactive and subjected to overdosing by DRT, whereas both the intact and denervated systems would be influenced abnormally by chronic stimulation of dopamine receptors by DRT. The two processes could independently or jointly contribute to an increased vulnerability to develop ICDs.

The notion that the ventral striatum, including the nucleus accumbens, integrates cortical and limbic inputs to regulate downstream structures involved in the inhibitory control of reward-related behaviors has gained considerable support in recent years (61, 103). A major unresolved question, however, is the precise role played by D_2_/D_3_ receptors in the inhibitory control of prepotent responses. One model posits that distinct populations of striatal cells mediate “Go” responses or “No-Go” responses (i.e., suppression of prepotent responses), the latter modulated by D_2_ receptors and the indirect striatal pathway (102). Networks tonically “overdosed” by D_2_/D_3_ receptor agonists (e.g., pramipexole and ropinirole) are thought to suppress reward-related learning by attenuating the effects of negative feedback on phasic dopamine release, thereby encouraging compulsive, perseverative behavior through the direct D_1_ receptor pathway. Although this model does not differentiate between different parts of the striatum (i.e., dorsal vs. ventral striatum), the distinction between tonic and phasic modes of dopamine release may be important in the context of ICDs in PD. Thus, tonic activation of D_2_ receptors is known to suppress PFC inputs to the ventral striatum and impair behavioral flexibility, whereas phasic activation of D_1_ receptors gates inputs to the ventral striatum from the ventral subiculum in the hippocampus (104). Therefore, excessive D_2_/D_3_ receptor activation by dopaminergic medications in PD may diminish inhibitory response control by the PFC and discourage flexibility of responding mediated within the direct and indirect striatal pathways. Interestingly, following 6-OHDA lesions of the dorsal striatum, pramipexole increased the preference of rats for uncertain (risky) rewards, similar to sham-operated control animals (87). Thus, excessive D_2_/D_3_ receptor activation may be a primary mechanism that generally promotes risk-taking impulsive behavior. Of interest, preferring D_3_ receptor agonists (pramipexole and ropinirole) appear to be more likely to cause ICDs in PD patients than relatively non-selective D_2_/D_3_ receptor agonists, such as bromocriptine (105), thereby suggesting that D_3_ dopamine receptors may be predominantly involved in DRT-induced ICDs; but for reasons that are not especially well understood, this provocation appears to be particularly apparent in some, but not all PD patients.

#### Impulsivity in PD: Beyond Dopamine

If progressive DA cell loss represents a major dysfunction in PD, other neurotransmitter systems, such as the serotoninergic and noradrenergic systems, which have been shown to contribute to impulse control (83, 106–109) are also affected in PD (110–112). Alteration of these other monoaminergic systems, which, in the case of noradrenaline, may precede the degeneration of dopaminergic neurons (111), are likely to contribute, independently, or in conjunction with dopaminergic denervation, to the development of impulsive behavior in PD. Indeed, the noradrenaline reuptake inhibitor atomoxetine, which decreases impulsivity and the associated vulnerability with compulsive behavior in rats (109), as well as the selective serotonin reuptake inhibitor citalopram, have recently been shown to improve response inhibition in PD, as assessed in a SSRT or a Go–No-Go task (113–115). The effect of these monoaminergic treatments on impulsivity in PD patients was associated with a restoration of the activity of the right inferior frontal gyrus and an improvement of its connectivity with the striatum (113–116).

Therefore, it appears that impulsivity in PD can be attributed, at least in part, to the degeneration of DA neurons and that it may facilitate the influence of DRT over the development of ICDs. But since impulsivity is not a unitary mechanism, but instead a complex multifactorial construct [e.g., Ref. (83)], associated with broad alterations within the corticostriatal networks and serotoninergic and noradrenergic dysfunctions, the neurobiological mechanisms contributing to the pathophysiology of impulsive behaviors in PD and their contribution to the development of ICDs may depend upon complex interactions between these systems, as it has been shown for compulsive drug seeking behaviors (96, 117–121).

However, impaired inhibitory control and underlying neurobiological substrates may not be the sole mechanisms that facilitate the development of ICDs upon DRT. Indeed, as postulated by the self-medication hypothesis of drug addiction, apathy, and anhedonia may contribute to the development of compulsive DRT use and associated ICDs within a negative reinforcement process (122–125).

## From Anhedonia to ICDs

### Phenomenology and Clinical Definitions of Apathy and Anhedonia

Alongside impulse control deficits, many PD patients develop apathy and anhedonia-related behaviors during the course of their disease (2, 11, 126, 127). Because the phenomenological and clinical description of anhedonia, apathy and mood disorders in PD has been extensively reviewed elsewhere [e.g., Ref. (11, 126, 128–130)], we will focus, here, on the elements that are of direct relevance for this review.

Apathy, previously defined as an absence or lack of feeling, emotion, interest, or concern, is currently viewed as a quantitative reduction of self-generated voluntary and purposeful behavior, resulting in low levels of activity, loss of socialization, and interest in sources of reinforcement (11, 131–133). The prevalence of apathy in PD varies from 13.9 to 70% depending on the population studied, the nature (instrument) of the assessment, and the period of investigation. Risk factor for apathy in PD are (i) being male (131, 134), (ii) lower education level (135), (iii) longer disease duration (136), (iv) severity of motor symptoms, and (v) executive dysfunction (135, 137, 138). However, psychiatric comorbidity greatly contributes to the vulnerability to apathy in PD since the highest prevalence is observed in PD patients with depression and/or cognitive dysfunction and apathy without depression and/or cognitive dysfunction ranges from 3 to 47.9% (138). Thus, although apathy is a clinical construct on its own with defined clinical sub-dimensions (see below), which is clearly distinct from anhedonia and depression (139–141), it frequently overlaps with anhedonia in PD (12, 130, 142, 143).

Anhedonia refers to a reduced ability to experience pleasure in response to stimuli usually perceived as rewarding (16). In PD, it has a prevalence of 5–46%, and it is significantly correlated with anxiety and depression (144). Anhedonia is not only a core symptom of depression in PD, but it has also been suggested to be a component of apathy, thereby contributing to the overlap that exists in PD between apathy and depression (145, 146). Thus, while anhedonia has been recognized as a symptom of both apathetic and depressive disorders (147), it may be, in the case of PD, a phenomenon secondary to these disorders [(130); but see Ref. (148)]. The recent subdivision of anhedonia into consummatory (inability to experience pleasure) and anticipatory (inability to link pleasure with a specific action) components may help to clarify the relationship between anhedonia and apathy, as well as depression in PD (142, 149). Specifically, as a motivational deficit, apathy in PD patients may be particularly related to the anticipatory subcomponent of anhedonia (142). Thus, a cross-sectional study performed in 95 untreated early-stage PD patients, reported apathy in roughly 19% of individuals, which was strongly associated with fatigue and anhedonia (143). These findings were further supported by the observation that PD patients experiencing clinically significant apathy also reported experiencing greater anticipatory anhedonia, thereby linking apathy to anticipatory, but not consummatory, hedonic deficits (150).

Because apathy is frequently associated with anhedonia, depression, and anxiety in PD [e.g., Ref. (12, 128, 130)], it has been suggested that these symptoms cluster into “hypodopaminergic behaviors” in order to facilitate the clinical management of such psychiatric manifestations in PD (18, 151, 152). These neuropsychiatric manifestations may, therefore, as we hypothesize here, share, at least in part, a common pathophysiological substrate.

At the clinical level, apathy has been conceptualized and operationalized as diminished goal-oriented behavior subdivided into three subtypes: emotional, cognitive, and auto-activation deficits, which are neurobiologically dissociable, in that they each depend upon a specific corticostriatal network (132). Thus, dysfunction of emotional processing, manifested as failure to associate affective and emotional signals, that would result in a reduced willingness to act (loss of will, loss of goal) or maintain ongoing action as well as the ability to evaluate the consequence of future action has been associated with alterations in the dopamine-controlled reward-related learning circuit encompassing the dopaminergic innervation from the VTA/SNc of the orbito-ventromedial PFC, ACC, amygdala, and ventral striatum (132). Similarly, dysfunction of cognitive processing, manifested as failure to manipulate goals, generates new rules or set shifting that would impaired action, has been associated with alterations in the dorsolateral PFC-striato-pallido-thalamo-cortical circuit. Finally dysfunction of self-activating processing, manifested as failure to self-generate behavior (contrasting with preserved response elicited by external-stimuli), reminiscent of a previously described behavioral syndrome termed “athymormia” (153, 154), has been associated with lesions affecting dorsal-medial PFC-supplementary motor area and ACC along with the limbic and associative territories of the thalamus and pallidum (132). This neuropsychological interpretation may be relevant to the neural mechanisms involved in the development of apathy and anhedonia in PD, which reflects in part the multidimensional facet of apathy with respect to factors such patterns of VTA vs. SNc denervation, disease duration, depression, and cognitive dysfunction. This may explain the failure of structural and functional studies to provide a unique anatomical pattern underlying apathy/anhedonia in PD [for review, see Ref. (11, 138, 155)].

### Mechanisms of Anhedonia and Apathy: The Case for Dopamine and Beyond

At the neurobiological level, anhedonia and apathy have been suggested to depend upon alterations of the dopaminergic systems in PD. Not only are apathy and anhedonia observed early in the disease, in *de novo* untreated patients, or even before the onset of motor symptoms (5, 52, 128, 156), but they are also displayed later on with the progression of dysexecutive syndromes (134). In this instance, they are likely related to the spread of synucleinopathy to the cortex (11, 157). Morever, apathy and anhedonia are also revealed as major side effects of STN-DBS (35, 48, 158).

#### Role of Hypodopaminergic States in Anhedonia and Apathy in PD

Especially at early stages of the disease or following STN-DBS, these hedonic and motivational deficits are alleviated by DRT, and particularly with D_2_/D_3_R agonists, such as pramipexole (142, 159, 160), thereby confirming that altered DA transmission may lie at the core of the pathophysiology of these non-motor symptoms. Consistently, several functional imaging studies in humans have reported positive correlations between the severity of apathy, depression, and anxiety in PD and the extent of the DA denervation in different regions of the corticostriatal circuitry including the ventral and the dorsal striatum and the prefrontal cortex, suggestive of a contribution of a denervation of both the nigrostriatal and mesocorticolimbic pathways to these hedonic/motivational deficits (152). This has been further supported by the recent evidence that apathy/anhedonia and anxiety in untreated early PD patients have been correlated to a decrease in DAT levels in the ventral and dorsal striatum, respectively (161, 162). In light of the recent evidence that a reduced striatal dopamine transporter availability predates the development of DRT-related ICDs (163), this study suggests that the striatal neurobiological underpinnings of apathy/anhedonia may represent a risk factor for the development of DRT-related ICDs.

Preclinical studies have confirmed this causal relationship between dopaminergic denervation and apathy/anhedonia. Apathetic- and anhedonic-like behaviors have been observed in MPTP-lesioned monkeys (56, 164, 165), and we have demonstrated that bilateral and partial DA lesion of the nigrostriatal system in rats, which caused no or mild motor deficits, dramatically impaired instrumental behaviors and induced depression- and anxiety-like behaviors (166–168). These motivational- and affective-related deficits following nigrostriatal DA denervations, replicated in other lesion-based rodent approaches, were shown to be fully corrected by DRT, and notably D_2_/D_3_R agonists [reviewed in Ref. (169)]. Taken together, these preclinical data strongly suggest that anhedonia-related behaviors in PD stem from the degeneration of SNc DA neurons (Figure 1).

#### Beyond Dopamine

However, preclinical studies have also indicated that affective-related deficits induced by 6-OHDA lesions in rodents also respond to serotoninergic receptor agonists or serotonin and/or noradrenaline reuptake inhibitors, therefore pointing to the implication other monoaminergic systems in the pathophysiology of apathy/anhedonia in PD (169, 170). This is consistent with imaging studies suggesting that mood impairments and fatigue in PD are also related to serotoninergic or noradrenergic dysfunctions (110, 112, 152) [but see Ref. (171, 172)]. Additionally, different and multiple dysfunctions of the corticostriatal circuits may account for the occurrence of hedonic-related deficits in PD, depending on the stage of the disease. For instance, DOPA-resistant forms of apathy, often related to cognitive decline, have recently been shown to be associated with atrophy of the ventral striatum in PD patients (173). In addition, and while it remains controversial, it is also suggested that STN-DBS may induce, or aggravate, apathy and depression, independently of DA, by interfering with the neuronal activity of the non-motor territories of the STN or by stimulation of nuclei or fiber tracts in close vicinity to the STN (169, 174–177). Therefore, and as already mentioned, the pathophysiology of apathy and related affective impairments in PD is multifactorial [see also Ref. (178)], and the implication of the nigrostriatal DA system may be limited to specific forms of anhedonia-related behaviors in PD, particularly those responsive to DRT.

### Apathy and Anhedonia: A Gateway for the Development of ICDs?

Irrespective of the precise heterogeneity of the pathophysiological mechanisms contributing to the development of anhedonia/apathy in PD, several studies have established a clear relationship between these emotional deficits, impulsivity, and ICDs. Indeed, depression and anxiety have been found to overlap with impulsivity in the same patients (9, 40). This is in agreement with the evidence that anhedonia tends to be more pronounced in PD patients with a gambling disorder (179) than in patients without, an interesting correlation already observed in non-parkinsonian pathological gamblers, who scored higher than controls on items of the Beck Depression Inventory (BDI) related to anhedonia and apathy (e.g., “loss of pleasure,” “loss of interest in other people,” or “loss of interest in sex”) (180). Similarly, Leroi et al. showed that both apathetic and ICDs PD patients presented a higher rate of anxiety and depressive symptoms than PD controls, suggesting that apathy and ICDs may share a common psychobiological substrate (14).

In this context, one may speculate that gambling in PD patients with high anhedonia/apathy reflects a compulsive behavioral process, initially aiming at self-medicating the anhedonic state that went awry. This is further supported by the evidence that compulsive DRT use is associated not only with ICDs but also with depression and anxiety (13, 71, 181, 182). Interestingly, depressive symptoms have been shown to increase in parallel with the development of new ICDs in PD patients (183), thereby suggesting that the more impulsive/compulsive behaviors the patient expresses as a consequence of DRT medication, the worse is his hedonic/emotional state, a conjecture highly reminiscent of the hedonic allostasis theory of addiction (124, 184). One of the predictions of this theory is that drug use in patients trying to self-medicate a hedonic deficit eventually further recruits between-system adaptations impinging on the stress and reward system, so that a strong emotional distress occurs upon withdrawal from the drug (123, 185, 186). This is exactly what was observed in 30 out 63 PD patients with a preoperative hyperdopaminergic profile (including, DDS, with compulsive use of medication, and various others ICDs) who developed apathy, depression, and anxiety with marked anhedonia and irritability following a marked reduction in their DRT after the initiation of STN-DBS (18, 158). Similar findings were observed in PD with ICDs in whom DRT were decreased (53), thereby suggesting that the specific vulnerability to ICDs brought by apathy/anhedonia may be related to an addiction-like state depending on the development of a compulsive attempt to self-medicate an internal distress, which will be worsen by a compulsive use of DRT (Figure 1).

## Conclusion: A “Two Head” Hypothesis of ICDs in PD

Taken together, the above analysis suggests that apathy/anhedonia/depression and impulsivity are dissociable symptoms along the same behavioral spectrum, but can overlap and contribute to ICDs in medicated PD patients.

On this ground, we propose in Figure 1 two different pathways underlying the development of ICDs in PD: one dependent on trait impulsivity and exacerbated by nigrostriatal DA denervation, which we hypothesize, interacts with DRT to facilitate the development of ICDs in specific PD individuals. The second pathway is dependent on the influence of the process on emotional, motivational, and hedonic states, resulting in a severe hedonic allostatic state, which vulnerable individuals may attempt to self-medicate through DRT and seek enhancement. These two putative psychobiological mechanisms should not, however, be viewed as contradictory. Indeed, along the progression of the disease, a complex combination of the degenerative process and of a premorbid weakness in inhibitory control are likely to occur and will act synergistically in order to create within or between disequilibria of the corticostriatal circuits, that will be exacerbated by DRT and notably D_2_/D_3_R agonists.

The iatrogenic hypothesis of ICDs suggests that repeated, pulsatile, and heightened stimulation of the DA mesolimbic system in PD with DRT induces maladaptive neuronal plasticity and hyperactivity of this system (12, 68, 70, 187), leading in turn to the development of aberrant reward sensitivity, as postulated by the incentive-sensitization theory of drug addiction (188). Although it allows interesting parallels between the mechanisms underlying addiction-like behaviors and dyskinesias in PD (9, 189), this hypothesis does not explain why only a subset of PD patients develop ICDs. Nevertheless, it would be possible to test the hypotheses developed in this article in longitudinal studies, in rodents as well as in PD patients, to investigate the interaction between different premorbid impulsivities (e.g., cognitive vs. motor), disease pathology, and the effect of dopaminergic medications. Delineation of these factors of vulnerability is a major challenge in the field to understand the pathophysiology and pathogenesis of ICDs and so better define and treat this comorbidity in PD.

## Author Contributions

J-LH, DB, and SC generated the hypotheses. J-LH, RM, JD, DB, and SC wrote the manuscript. DB worked on the figure.

## Conflict of Interest Statement

The authors declare that the research was conducted in the absence of any commercial or financial relationships that could be construed as a potential conflict of interest.
